# Supplementary material for: Chromothripsis during telomere crisis is independent of NHEJ, and consistent with a replicative origin
Source: Genome Res. 2019 May;29(5):737–49. doi: 10.1101/gr.240705.118 (PMC6499312; doi:10.1101/gr.240705.118)
Supplement: Supplemental Material [file supp_gr.240705.118_Supplemental_file_1.zip › contigs/annotated_contigs/DB56/contig.2.DB56_length_426_mean_cov_5.19718309859.docx]

**DB56_length_426_mean_cov_5.19718309859**

```
TGCCTTAGCCCCCCGAAGTGCTGGGATTACAGGTGTGAGCCACCGCAGCCAGCCAAATTTAGACATTGCTTAACTAAATAACGGGTTCA
 >chr7:138148986-138149183 + E=5e-101
TTTGATCAAACCAGTTCTTCATTTTCCTACTGAATTTGTGAGTACAGGCATCACAAGTCAAGTAATATCTATTACACAAAGGTTAAATA

GGTAGTTGGGATGGA|GAAC|ATGTATTGCAGCTCACTTTTCTAGCTCAGCTCCTGGCATGTGGTTGGTGCCAACCTGGTGGGCCATAT
                >chr11:44628979-44629210 - E=1e-121
TTATTACTGTGAGATGGTGAATCACATAGGGTGGACTGCCCAAGATCCCTGCCGAGGCGACCCGCTTCTGAGTCATAGAAACCGGCTCA

CTGGGGCCTGGTTCTAATCCACCCTGATTATATCACTAACTAGGTCCTAAAGAAGCTCAGTGCCATAGGA|AA
```
